# Fundamentals of FAIR biomedical data analyses in the cloud using custom pipelines

**DOI:** 10.1371/journal.pcbi.1013215

**Published:** 2025-07-02

**Authors:** Seth R. Berke, Kanika Kanchan, Mary L. Marazita, Eric Tobin, Ingo Ruczinski

**Affiliations:** 1 Department of Biostatistics, Johns Hopkins Bloomberg School of Public Health, Baltimore, Maryland, United States of America; 2 Division of Allergy and Clinical Immunology, Johns Hopkins School of Medicine, Baltimore, Maryland, United States of America; 3 Genomics and Precision Health Section, Laboratory of Allergic Diseases, The National Institute of Allergy and Infectious Diseases, Bethesda, Maryland, United States of America; 4 Center for Craniofacial and Dental Genetics, Department of Oral and Craniofacial Sciences, School of Dental Medicine, University of Pittsburgh, Pittsburgh, Pennsylvania, United States of America; 5 Initial Testing Department, Ethos Laboratories, Newport, Kentucky, United States of America; 6 Department of Biology, University of Cincinnati, Cincinnati, Ohio, United States of America; SIB Swiss Institute of Bioinformatics, SWITZERLAND

## Abstract

As the biomedical data ecosystem increasingly embraces the findable, accessible, interoperable, and reusable (FAIR) data principles to publish multimodal datasets to the cloud, opportunities for cloud-based research continue to expand. Besides the potential for accelerated and diverse biomedical discovery that comes from a harmonized data ecosystem, the cloud also presents a shift away from the standard practice of duplicating data to computational clusters or local computers for analysis. However, despite these benefits, researcher migration to the cloud has lagged, in part due to insufficient educational resources to train biomedical scientists on cloud infrastructure. There exists a conceptual lack especially around the crafting of custom analytic pipelines that require software not pre-installed by cloud analysis platforms. We here present three fundamental concepts necessary for custom pipeline creation in the cloud. These overarching concepts are workflow and cloud provider agnostic, extending the utility of this education to serve as a foundation for any computational analysis running any dataset in any biomedical cloud platform. We illustrate these concepts using one of our own custom analyses, a study using the case-parent trio design to detect sex-specific genetic effects on orofacial cleft (OFC) risk, which we crafted in the biomedical cloud analysis platform CAVATICA.

## Introduction

Cloud computing has long held the theoretical promise of facilitating large-scale bioinformatic analyses due to its ability to house and assess big data [1]. However, as biomedical data initially expanded in the cloud, many datasets remained siloed within repositories, often lacking standardized metadata and data-sharing frameworks. This made integrative and innovative analyses across repositories a challenging task. Once academia, industry, and publishers recognized the widespread incompatibility in the biomedical data landscape, they jointly established the Findable, Accessible, Interoperable, and Reusable (FAIR) data principles to enhance data access and sharing [2]. While there is still room for improvement, FAIRness has been largely successful, as many data resources have embraced the principles, and efforts to further FAIRify data continue to grow [3]. Concurrently, biomedical computing in the cloud made large strides, with platforms such as Seven Bridges [4], Terra [5], and Galaxy Cloud [6] helping to improve interoperability between repositories such as the database of Genotypes and Phenotypes [7] (dbGaP), the Gabriella Miller Kids First Foundation [8] (GMKF) , and large-scale biobanks such as the All of Us Research Program [9], presenting the opportunity for researchers to migrate to the cloud to conduct novel, integrative analyses. Although increased data ecosystem interoperability alongside accompanying analytic platforms would seemingly imply expedited biomedical discovery, researchers have faced significant friction in the cloud, especially for those interested in carrying out non-standard, specialized analyses.

Biomedical cloud data analysis platforms, also called data commons [10], are providers that aim to facilitate analysis by centralizing data storage and analytic tools to carry out workflows in the cloud. Many standard software packages (such as VCFtools [11] for analyses of sequencing data) are commonly made available by such providers. However, due to the nonstandard nature of custom analyses, many tools (defined as distinct processing steps in an overall workflow) needed for their execution are often not pre-installed by the platforms [12]. This has become especially true as providers continue to differentiate into niches with certain data and tools, leaving no singular platform versatile to every analysis. Thus, one platform that may include a needed specialized tool for a given pipeline may be incompatible with the rest of a researcher’s workflow or data [12], often leaving investigators reliant on local or high performance computing (HPC) clusters to carry out custom steps. The mixture of local, HPC, and cloud architecture to execute nonstandard workflows has led to an unwieldy technique where researchers scatter to adapt workflows (or portions of workflows) to architecture-specific complexities and gather results back together post-hoc. The issue pervades biomedical research; thus, the FAIR Principles for Research Software (FAIR4RS) have been established to improve the landscape of biomedical software analysis by making analytic tools more accessible and shareable [13]. Therefore, there is an urgent need for streamlined approaches to build comprehensive, customized workflows in the cloud that ensure the accessibility and implementation of specialized analytic tools. The magnitude of the potential benefits can be understood when for example considering the Summary Statistics of dbGaP Data webpage, which shows the cumulative counts of approved data access requests approaching 100,000. Data access requests are commonly submitted to test scientific hypotheses not considered among the respective studies’ primary aims, and often require specialized software to carry out the investigation.

**Fig 1:**
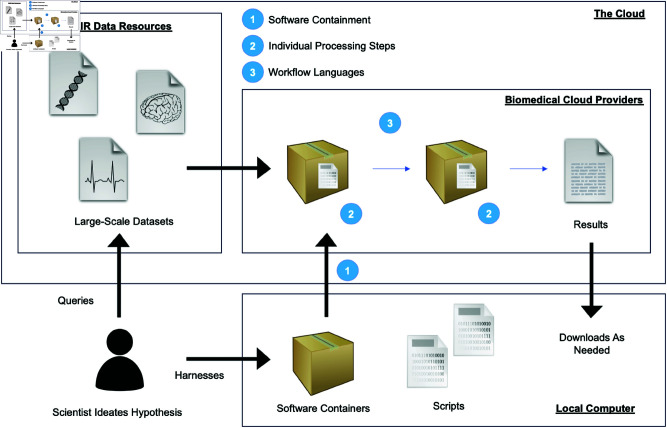
A schematic of the fundamental concepts and flow to carry out custom cloud pipelines. A walk-through video of the diagram is hosted in the tutorial videos.

In this manuscript we offer guidance to enable investigators with basic computational skills to migrate, develop, and conduct their custom analyses in the cloud based on three fundamental principles we believe are essential to any efficiently implemented cloud-based pipeline, particularly when designing custom analyses: packaging computational environments into software containers, generating code for individual processing steps, and weaving steps into analytic pipelines using workflow languages (Fig 1). These principles apply universally, regardless of the biomedical cloud platform a researcher chooses. Popular platforms such as Terra, Galaxy Cloud, and Seven Bridges can become more accessible we believe when following those principles. Though the principles we discuss apply in general, the example presented in this manuscript most directly aligns with the Seven Bridges ecosystem, including the Cancer Genomics Cloud (CGC), Biodata Catalyst (BDC) and CAVATICA. Specifically, we detail and illustrate the respective principles using a CAVATICA workflow example on FAIR data from the National Institutes of Health’s (NIH) Common Fund GMKF Pediatric Research Program, and offer narrated videos on a companion website that help to demonstrate how to implement the respective steps in CAVATICA. We expect our approach to remain platform, software, and programming language agnostic as the volume of high-throughput data residing in the cloud continues to grow.

## Fundamental 1: Package custom computational environments into software containers

Software containment is the bridge between local computation and the cloud; it is what allows for scripts created with diverse programming languages and scientific packages to be executed outside of the confines of the local machine or cluster within which it was developed. When an analytic pipeline contains scripts written with different programming languages and packages, it is considered to be “polylingual” and must be handled appropriately on the cloud [14]. To account for the heterogeneity of languages and packages across processing steps in a given pipeline, a researcher must assess the runtime environment of each individual step by identifying its required programming language, packages, and files, alongside other environmental configurations. Creating a software container for each unique computational environment is critical, as it allows the remote servers to know which languages and packages to invoke for the execution of each script.

Containerization platforms such as Docker [15] or Apptainer (former Singularity) [16] are programs designed to package the dependencies and environmental settings of a given script into isolated, portable units. Traditionally, virtual machines (VMs) were used to run specialized computational environments, but require heavy computational resources and have largely in recent years been replaced by containers [17]. While there are many platforms, Docker arguably has become the industry standard [18]. It streamlines container development with three key components: the i) Dockerfile, which holds the instructions to construct a ii) Docker Image, which is a snapshot of the software and dependencies that will be run in the iii) Docker Container, which is the associated executable environment. Docker Images are immutable, ensuring enduring reproducibility in computational research [19]. Here is the typical structure of a simple Dockerfile:

FROM baseimage
# to inherit from a pre-existing image
COPY ./files /path/in/container
# to copy in needed files
RUN <command_to_install_package>
# to add a computational package

**Fig 2:**
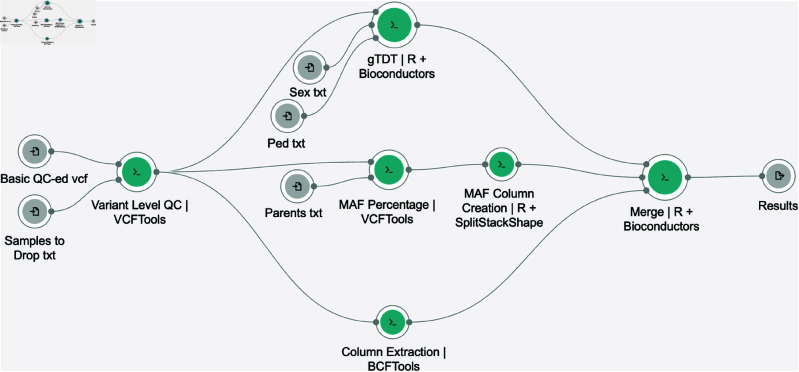
A schematic of the gTDT analysis pipeline in the workflow visual editor. Teal colored circles represent CAVATICA tools. Grey circles represent input and output files. Lines indicate the connection between files and tools.

Many biomedical cloud platforms directly integrate with Docker so scientists can port their computational environments. In our case study, we harnessed CAVATICA’s Docker Registry to send several distinct environments to the cloud with Docker as a part of our custom polylingual workflow. An example demonstrating a custom computational environment is the Dockerfile for our gTDT | R + Bioconductors step (Fig 2). This step was written using the R Programming Language and required several add-on packages, including the Bioconductor [20] package $\text{tri}\vec{o}$ to test for sex differences in genetic effect sizes underlying OFC risk using a genotypic transmission disequilibrium test (gTDT) suitable for case-parent trio designs [21,22] (see S1 Text for more scientific and statistical details). Here is the Dockerfile we created:

FROM bioconductor/bioconductor_docker:devel-amd64
RUN R -e "install.packages(’data.table’)"
RUN R -e ’BiocManager::install("VariantAnnotation",
force = TRUE)’
RUN R -e ’BiocManager::install("trio")’

To port the environment to the cloud, there are additional steps to be performed locally which first transform the Dockerfile into a Docker Image and then send it to a designated location. Here, we are sending our Docker Image to a repository in CAVATICA named “name”. We also demonstrate this step in our supplementary videos linked from our GitHub page. While this brief explanation is Seven Bridges-specific, each platform has a similar method to port and utilize software containment.

docker login pgc-images.sbgenomics.com
# logs in to CAVATICA
docker build -t name . –platform="linux/amd64"
# builds image from Dockerfile
docker tag name pgc-images.sbgenomics.com/username/name
# proper name for transport
docker push pgc-images.sbgenomics.com/username/name
# ports it to the cloud

Additionally, Docker is open source and published Docker Images from the software development and scientific communities can be freely pulled from DockerHub. Thus, before crafting individualized containers, researchers are advised to check whether the runtime environment for a given script has already been “dockerized”. Importantly, Docker is also modular, meaning an image with only parts of the required dependencies for a given step can have layers added on top to fulfill all requirements [18]. In our application, we used several DockerHub images directly and built on top of others. Specifically, we directly used DockerHub Images for VCFTools and BCFTools. However, there was no pre-existing Docker Image with the Bioconductor package trio. Instead, as shown above, we added multiple layers on top of the Bioconductor base image with each “RUN” command. The full list of all software containers used in our study is listed on our GitHub page.

Across any biomedical cloud provider, software containment is what allows for an individual processing script with a certain computational environment to run in the cloud. However, it is important to note that specific providers integrate with certain methods. Docker remains the standard for environments such as Seven Bridges and Terra, whereas Galaxy Cloud supports both Docker and Apptainer. As explored further in the Discussion section, public Docker containers can raise security concerns, particularly because they require elevated system privileges to operate. It is therefore essential for users to thoroughly vet any container they intend to use. In contrast, Apptainer does not require the same high level system access (but still should be vetted). Ultimately, the choice between Docker and Apptainer depends on the platform and the user’s priorities regarding functionality, compatibility, and security.

## Fundamental 2: Create individual processing steps

Individual processing steps are code scripts that constitute the building blocks of an overall custom pipeline, and once software environments for each script are containerized and sent to the cloud, scripts can be placed inside their proper containers for execution. Therefore, each step in a pipeline has two key components: 1) a software container with its unique computational environment and 2) a script to process data. A script can be of any size and complexity, but all share the quality of having an input, performing some sort of processing, and outputting meaningful information for the analysis. For example, a single bash line executing a simple filter command constitutes a processing step, as does a complex Python script. Members of the scientific community utilizing biomedical cloud platforms often publish scripts that may be useful for other workflows, and researchers can choose to incorporate them into their own workflows. However, when researchers need a processing step not pre-published on the platform, they will need to construct their own.

In our case study and in the online videos we demonstrate how to construct an individual processing step in CAVATICA. The example directly applies to the platforms within the Seven Bridges ecosystem, but the underlying concept is platform-agnostic. Here, we first explain the processing steps in CAVATICA and then discuss how tool creation is implemented on other major platforms such as Terra and Galaxy Cloud.

In Seven Bridges and thus in CAVATICA, an individual processing step is referred to as a “tool”. Each tool in a pipeline is depicted graphically in the visual editor as a teal circle with input and output ports (Fig 2 and Fig A in S1 Text). To customize processing steps for specific analyses in CAVATICA, investigators first name the tool which gives them access to the editor for creation. The tool editor has a fill-in interface to craft commands and specifications. Researchers must select a Docker Image that contains the proper software dependencies, construct an executable bash command, include file scripts when needed, and configure inputs and output specifications. There is also an option to save output and error logs generated during runtime for debugging. The tool editor implements software development standard practices that any integrated development environment (IDE) would, such as version control similar to Git, debugging aid and error logs, and extensive documentation opportunities. As an example we again highlight the tool that executed our custom gTDT analysis, termed gTDT\,{|R+Bioconductor}. The script gTDT.R, along with the scripts of each of our implemented tools, is available on our GitHub page. Each input and output file in the bash command corresponds to a grey circle in the visual editor, for example, Ped.txt is an input for the gTDT\,{|R+Bioconductor} tool (Fig 2). Once constructed, the tool’s command line code that is executed by the cloud environment reads:

Rscript ./gTDT.R input_ped.txt input_sex.txt input_vcf.
txt > stdout.out

Galaxy Cloud, similar to platforms under the Seven Bridges umbrella, takes a graphical, user-friendly approach. Individual processing steps are also called tools and can be configured through a web-based interface. Terra takes a slightly different approach where individual steps are typically defined within a single workflow (see Fundamental 3 for details on workflows) without using a graphical user interface (GUI). Each individual processing step in Terra still harnesses an individual docker container with the necessary dependencies.

Importantly, the modularity presented by individual processing steps comprising cloud pipelines presents the opportunity to combat the often costly tweaking of bioinformatic pipelines (“tinkering”) in the cloud. To reduce cost while testing our workflow, we leveraged a few computational principles: i) verifying the efficacy of each standalone step, ii) conducting testing with minimally sized data, and iii) leveraging memoization, an optimization technique for the re-running of computational jobs that re-uses results for the aspects of a task that remain unedited [23]. Through this approach, we streamlined tool development and validation with minimal pricing. While standalone step by step execution can help with development, running an entire analysis in this way creates inefficiencies: intermediate, derived files clutter storage and the execution of many tasks takes significant manual labor and time. Therefore, crafting steps together into a polylingual workflow becomes necessary for efficient research.

## Fundamental 3: Weave together processing steps with workflow languages

Workflow languages are implemented to weave together distinct steps into an overall analytic pipeline. There are several different workflow languages popular in the scientific community such as Nextflow, Workflow Description Language (WDL) and Common Workflow Language (CWL), each with differences in extendability, readability and other attributes [24]. They have become increasingly popular in recent years as a way to address the execution of polylingual analytic pipelines by abstracting the management of computational environments (software containment), the movement of data between individual processing steps and the scheduling of job execution with proper resource allocations [24]. While complete workflow language literacy is necessary to harness every possible biomedical cloud provider, certain providers, namely those under the Seven Bridges domain and Galaxy Cloud, lower the entry point to workflow weaving through the implementation of a visual drag-and-drop editor, such that workflows can be crafted without the explicit writing of the tedious syntax of the underlying code. We note that this visual editor especially holds the capability of allowing less experienced computational researchers to leverage the utility of workflow languages. For instance, in CAVATICA, researchers can add custom made or publicly available tools to the editor and connect tools through their input and output ports, creating lines between tools that represent the flow of files within the pipeline (Fig 2 and Fig A in S1 Text).

Workflow languages all share the common goal of orchestrating complex pipelines to handle large-scale data across processing steps created from heterogeneous computational environments. Different options however also translate to differences in ease of development, portability, and robustness [24]. When choosing a platform, the user generally also inherently chooses the cloud provider and the workflow management system. For instance, Seven Bridges (which we used for our example) harnesses CWL, often cited as a low-ease-of-use workflow language [25], but due to the GUI native to Seven Bridges we found the entry point to be manageable. Terra implements WDL and Galaxy Cloud implements an in-house language that also leverages a GUI interface. As mentioned in Fundamentals 2, Terra takes a slightly different approach as workflow steps are typically created concurrently within a single WDL script, allowing them to be seamlessly chained together as part of one coherent workflow. This structure promotes reproducibility and efficiency by capturing the full computational logic in one place. To maintain modularity, users can choose to separate each analysis step into its own individual workflow. However, once each step is validated, users will want to construct an overarching WDL script to weave together these steps.

Workflow editors should also maintain software development standards such as version control, documentation, and extensive debugging. Leveraging memoization, a routine optimization technique that caches the results of previously computed function calls, is particularly valuable in this context. By storing outputs from unchanged workflow steps, researchers can avoid redundant computations, significantly reducing runtime and cloud resource costs. This approach ensures that only modified or newly introduced steps in a pipeline are re-executed, enhancing efficiency and scalability in cloud-based workflows. In all, runtime environments are ported to the cloud through software containment, then individual processing steps are constructed using these containers, and finally workflow languages weave together each step into a complete, cloud-executable custom analytic pipeline.

## Discussion

In this manuscript we presented the fundamental concepts to create specialized biomedical cloud analyses and illustrated the approach by our own use case on CAVATICA. As discussed throughout the manuscript, our approach is also applicable to other platforms and we hope that the guidance offered in this manuscript will enable other investigators with basic computational skills to implement and execute their own analytic pipelines in the cloud. In particular, we believe our approach adheres to the FAIR data principles by promoting data reuse for new biomedical discovery.

The development process – once understood – was quick in its implementation and efficient in its execution. We used standard software development approaches for code development and porting. Version control was especially critical in saving progress for each modular step; we found that the ability to return to a specific time point during development was both useful and an ease of mind when a code edit introduced a bug into the workflow. We implemented the discussed methodology to “tinker” without excessive time or cost by periodically validating single steps or the entire pipeline, utilizing a minimally sized dataset. This allowed us to re-run tools during the debugging process. Leveraging the CWL workflow system and the automatic job scheduler allowed us to carry out complex workflows with ease, keeping the costs low for a highly scalable analytic pipeline.

Developing this approach involved a somewhat steep learning curve and overcoming some hurdles, in part due to a lack of manuals, documentation and guidance we encountered at the time of our implementation. Developing cloud computing with a polylingual workflow logic requires a solid knowledge of the fundamentals for software containment, individual processing step creation, and workflow language systems. A new user might become frustrated when encountering a lack of documentation about the computing infrastructure and the file system. In particular with our videos on the companion website, we hope we will be able to flatten that learning curve for other investigators. Once understood, we believe the implementation of any study can become very much streamlined. Our goal was to lower the entry point for cloud research by empowering researchers from diverse scientific backgrounds to craft cloud-based custom pipelines and to investigate novel hypotheses with varied datasets. Readers of our manuscript might also find additional documentation for workflow generation helpful, which was recently made available by major platforms such as Terra, CAVATICA, and Galaxy and specific examples for these platforms are available online as well (here, here and here).

Once users select a provider and develop a custom workflow, they have several deployment options. The most seamless approach is to deploy the workflow on a cloud instance from the same provider that created it, which typically employs either Amazon Web Services (AWS) or Google Cloud Platform (GCP). Transferring workflows between providers can be challenging due to provider-specific terminology, often leading to provider “lock-in”. [12]. However, within the Seven Bridges ecosystem, shifting workflows across platforms like CGC, CAVATICA, and BDC is straightforward. Additionally, Seven Bridges offers the ability to easily export workflows for local computation when necessary. That said, in line with the goals of cloud adoption, the data is intended to remain in the cloud. Increasingly, partnerships such as the recently announced Galaxy and Bioconductor Community Conference are being formed between biomedical software groups and cloud providers to facilitate the custom analysis of large-scale data directly in the cloud.

After deployment, scientific work should be reproducible. Despite this, reproducibility remains a key challenge for cloud-based custom pipelines, particularly when workflows are executed within proprietary ecosystems such as CAVATICA. While we have made our pipeline (divided into its individual tasks) available on GitHub, true reproducibility of our work and many custom cloud workflows is inherently constrained by several factors. First, workflows implemented using a specific workflow language are not always easily portable outside the environment it was developed in, as cloud-based execution often relies on platform-specific configurations. Although many workflows are designed for interoperability, including CWL, adapting workflows for execution in different environments requires additional effort and standardization, which is an ongoing challenge in the field. Additionally, controlled-access data introduces another layer of complexity when considering true reproducibility of cloud-based pipelines, which often rely on biological raw data that may introduce privacy concerns when no governance oversees the data’s distribution. As a result, full reproducibility is limited to researchers who can obtain similar datasets under identical access policies. However, emerging efforts have sought to enhance the potential for complete reproducibility of biomedical cloud-provider based work. For instance, all Seven Bridges platforms maintain a Public Gallery where users can publish their functional tools and workflows for others in the ecosystem to pull and implement for their own analyses. Additional efforts such as Dockstore and WorkflowHub are paving the way for improved portability and interoperability for custom cloud-based analyses. We note that future developments in interoperability and standardization will be critical for making cloud-based workflows more FAIR.

Metadata standardization is also essential to ensure reproducibility and to fully leverage the potential of large, multi-source datasets. In our case study for example, the absence of pedigree files made it initially impossible to perform transmission disequilibrium tests as the available Variant Call Format (VCF) files do not contain information on familial relationships to readily identify parents and probands. Besides familial relationships, pedigree files also provide the sex of the participants, which was needed for our analysis investigating sex-specific effects. While in our situation one could argue that familial relationships could be determined with high reliability from identity-by-descent calculations and sex could be determined from X chromosome genotypes, we ultimately had to trouble the principal investigator of the study for the pedigree files, an approach we do not think is in agreement with the FAIR principles. These types of challenges are not uncommon and have plagued the field for a long time. In 2001, the MIAME (Minimum Information About a Microarray Experiment) [26] guidelines were introduced to outline the minimum information that should be included when describing and sharing data from a microarray experiment, which in particular specified standardized metadata requirements. Similar guidelines based on these MIAME principles were subsequently published for many other technologies and fields [27–33]. Enforcing such standards we believe will support the FAIR principles and empower researchers to confidently access and reuse data for their own analyses.

Beyond reproducibility and metadata concerns, it is also critical to mention security risks while working with biomedical data. Due to the fact that our approach also supports restricted-access datasets, such as the dataset we utilized in our case study, it is essential for researchers to consider the security risks associated with high-level system access. While public containers and scripts enhance reproducibility and offer convenience, they can also introduce risks if not properly vetted. This is especially true when containers, like those in Docker, require elevated system privileges, potentially exposing systems to unauthorized data access, unintended changes to settings, or malicious code execution. While cloud platforms offer safety measures such as isolated VMs that are disconnected from personal systems or institutional clusters, vulnerabilities can still arise if system access is not carefully managed. Apptainer provides a more secure alternative in many computing environments since it does not require elevated privileges [34]. Regardless of the platform, it is crucial to control system access and carefully manage how data and workflows are executed to prevent the unintended exposure or loss of sensitive information.

Additionally, as with the adoption of any new methodology, cost considerations play a significant role. Similar to many HPC systems, cloud costs are commensurate with the chosen instance type. Users can choose from a wide range of instances to most optimally fit the CPU / RAM costs they need. One notable financial advantage of cloud computing is storage efficiency. Through the Global Alliance for Genomics and Health’s (GA4GH) [35] Data Repository Service, available on all platforms discussed in this manuscript, users can directly access, analyze, and interpret data from its original source on the cloud without need for duplication that leads to storage costs. However, to fully realize these cost savings, users must actively manage and trim data derivatives and intermediates as needed. When properly maintained, cloud computing offers significant storage benefits and may offer cost savings as well. Billing groups and cloud-resources are provider-specific however, and users will have to work within the platform of their choice to obtain or supply the necessary funds to execute their pipelines.

In all, with the abundance of FAIR data and increased interoperability between datasets and types from diverse data resources, we are enthusiastic about the potential for novel scientific hypotheses to be raised and tested within many large datasets now cloud-accessible. We believe there could be a real paradigm shift especially for non-standard analyses, which are typically carried out in-house on local machines or HPC computing. We hope that the guidance we offer will be useful for investigators who wish to develop specialized analyses in or migrate specialized analyses to any biomedical cloud platform. We do appreciate that researchers are typically much more familiar with the details of their local HPC environment compared to cloud computing options, which naturally raises the question about research productivity, an issue we aim to alleviate in this manuscript. As the scale and cloud-accessibility of biomedical data continue to expand, we believe there are increasingly compelling arguments for cloud adoption, particularly due to storage considerations as described above. Cloud platforms enable researchers to bring computation to the data, minimizing the need for potentially costly and inefficient data replication across systems. A prominent example is the TOPMed consortium exchange area, which is currently (April 2025) in transition from dbGaP to BDC, with this particular cloud being designated as the only place to carry out future analyses as data downloads will no longer be possible. We hope our manuscript is a timely contribution for users making similar transitions to the cloud.

## Supporting information

S1 Text Supporting text with embedded additional supporting materials. Containing 3 supporting sections describing the scientific background of the case study, the technical background and quality control conducted, and the statistical background, plus one supporting figure and additional references.
(ZIP)
