# Supplementary figures and images for: Fundamentals of FAIR biomedical data analyses in the cloud using custom pipelines
Source: PLoS Comput Biol. 2025 Jul 2;21(7):e1013215. doi: 10.1371/journal.pcbi.1013215 (PMC12221167; doi:10.1371/journal.pcbi.1013215)

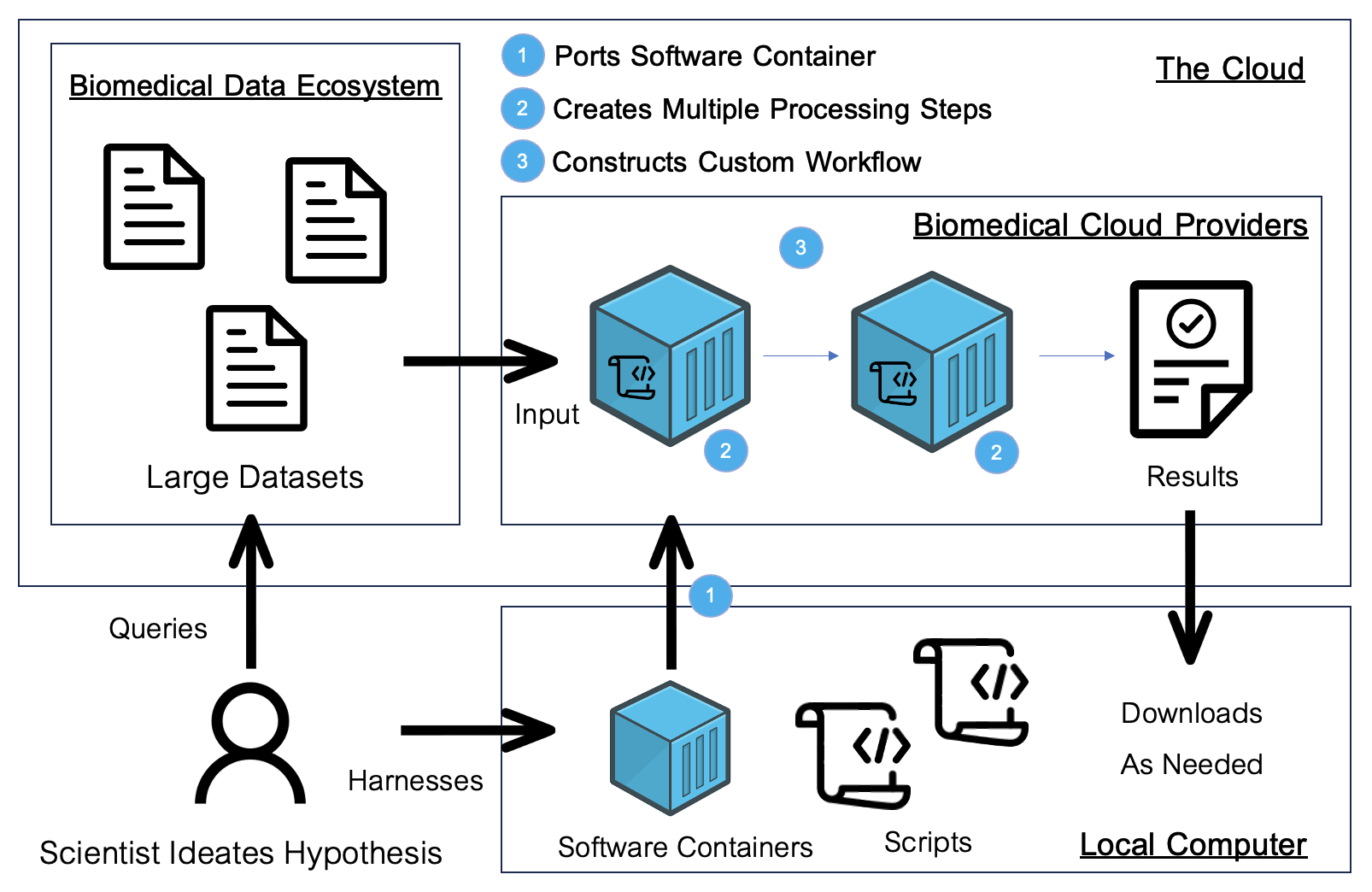

Supplement: S1 Text — Containing 3 supporting sections describing the scientific background of the case study, the technical background and quality control conducted, and the statistical background, plus one supporting figure and additional references. (ZIP) [file pcbi.1013215.s001.zip › figs/Bioinformatic_Flow.png]

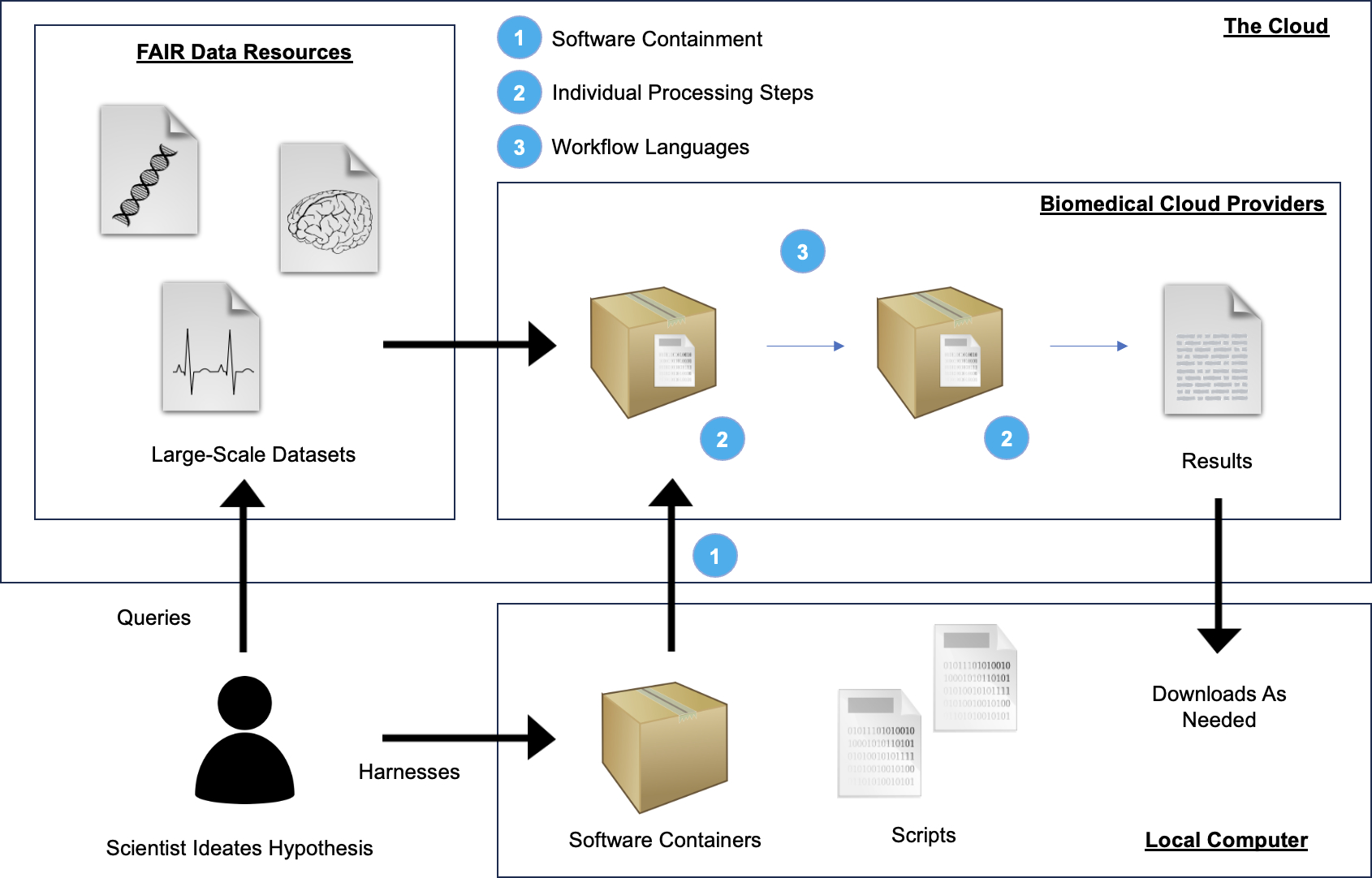

Supplement: S1 Text — Containing 3 supporting sections describing the scientific background of the case study, the technical background and quality control conducted, and the statistical background, plus one supporting figure and additional references. (ZIP) [file pcbi.1013215.s001.zip › figs/Fig1.png]

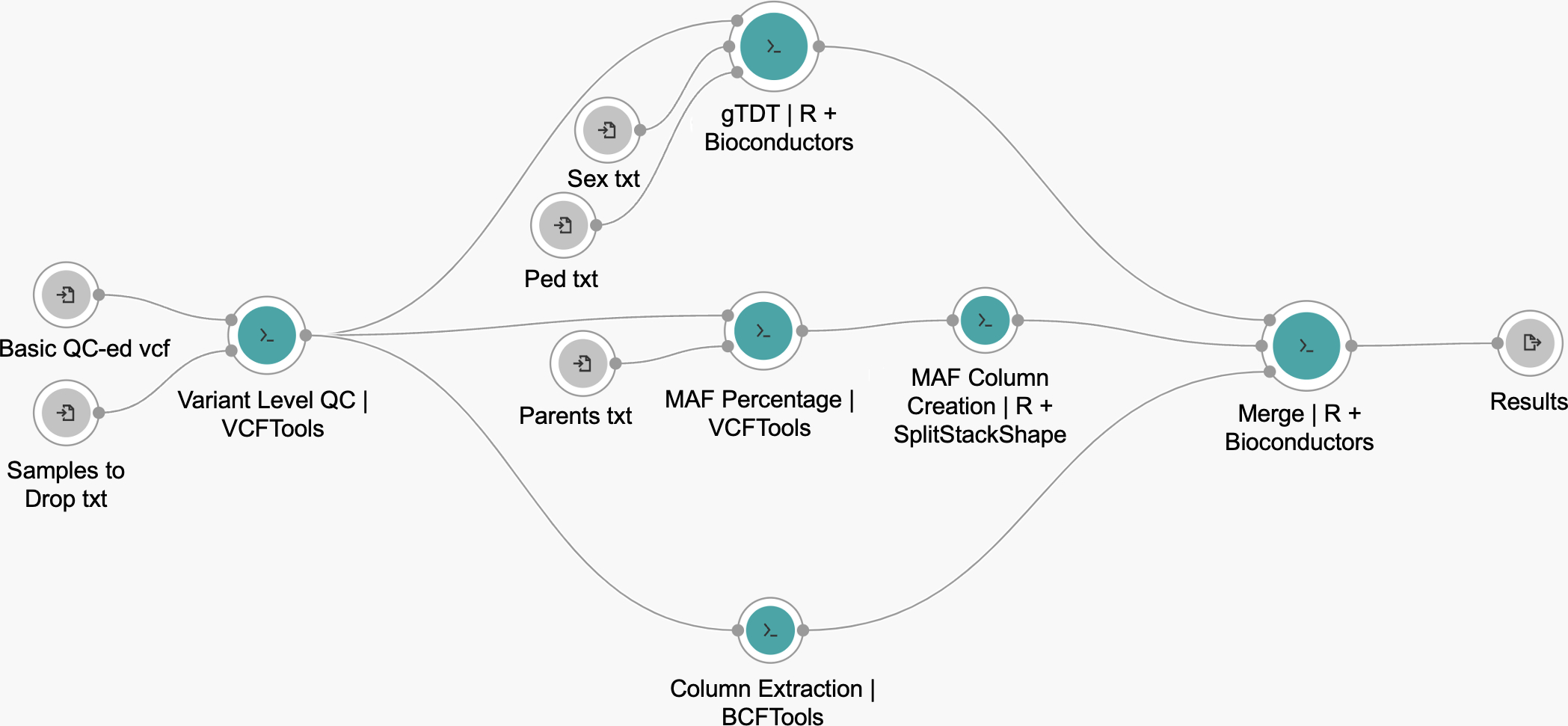

Supplement: S1 Text — Containing 3 supporting sections describing the scientific background of the case study, the technical background and quality control conducted, and the statistical background, plus one supporting figure and additional references. (ZIP) [file pcbi.1013215.s001.zip › figs/Fig2.png]

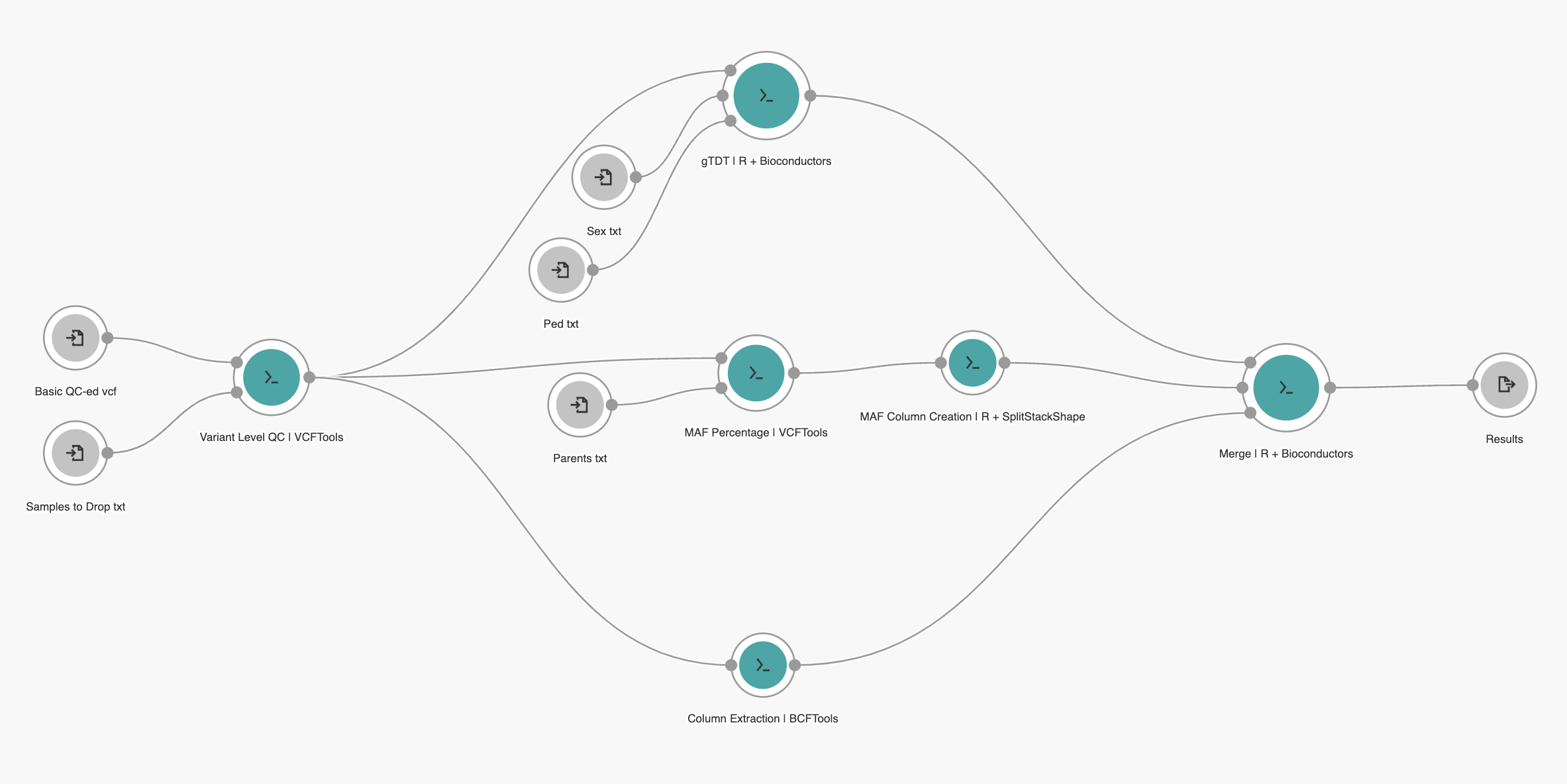

Supplement: S1 Text — Containing 3 supporting sections describing the scientific background of the case study, the technical background and quality control conducted, and the statistical background, plus one supporting figure and additional references. (ZIP) [file pcbi.1013215.s001.zip › figs/gTDT.CWL.png]
